# TAFRO syndrome requiring combined IL 6 and IL 1 inhibition: a case report

**DOI:** 10.3389/fimmu.2025.1729525

**Published:** 2026-01-06

**Authors:** Ahmad Wael Sultan, Eva Grießhammer, Carl Hinrichs, Lars Trenkmann, Ilske Oschlies, Leif Gunnar Hanitsch, Hildrun Haibel, Thomas Schneider, Rasmus Leistner

**Affiliations:** 1 Department of Gastroenterology, Infectious Diseases and Rheumatology, Charité Universitätsmedizin Berlin, Corporate Member of Freie Universität Berlin and Humboldt Universität zu Berlin and the Berlin Institute of Health (BIH), BIH Center for Regenerative Therapies, Berlin, Germany; 2 Department of Nephrology and Intensive Care Medicine, Charité Universitätsmedizin Berlin, Corporate Member of Freie Universität Berlin and Humboldt Universität zu Berlin and the Berlin Institute of Health (BIH), BIH Center for Regenerative Therapies, Berlin, Germany; 3 Department of Hematopathology and Lymph Node Registry (Division of Pathology), Universitätsklinikum Schleswig-Holstein, Kiel, Germany; 4 Institute of Medical Immunology, Charité-Universitätsmedizin Berlin, Corporate Member of Freie Universität Berlin and Humboldt Universität zu Berlin and the Berlin Institute of Health (BIH), BIH Center for Regenerative Therapies, Berlin, Germany

**Keywords:** case report, combined IL-6 and IL-1 inhibition, idiopathic multicentric Castleman disease, iMCD, TAFRO syndrome

## Abstract

**Introduction:**

TAFRO syndrome is a rare and severe variant of idiopathic multicentric Castleman disease (iMCD). The name-giving presentation is a combination of thrombocytopenia, anasarca, fever, reticulin fibrosis/renal dysfunction, and organomegaly. The disease’s complex but unspecific presentation shows overlapping features with hyperinflammation syndromes of infectious, malignant, or autoimmune origin. Here, we present a case of a young patient with iMCD TAFRO refractory to targeted IL-6 inhibition.

**Case description:**

A 21-year-old previously healthy male developed progressive systemic inflammation with fever, persistent generalized lymphadenopathy, bicytopenia, polyserositis, and renal failure. Infectious diseases and oncological and immunological workup within the first 6 months after symptom onset yielded an unclear hyperinflammatory syndrome. Subsequently, the patient was treated with IL-1 inhibition resulting in partial symptom relief. However, after a flare, the repeated lymph node histopathology showed immunohistochemical features of iMCD and the diagnosis iMCD TAFRO was established. The patient was then additionally treated with IL-6 inhibition. An attempt to switch to monotherapy with IL-6 inhibition resulted in another flare-up of the disease. This demonstrated the need for continued combined IL-6 and IL-1 inhibition. Under this combination therapy, the patient showed complete and stable remission, which persisted even after 12 months of follow-up.

**Conclusion:**

This case highlights the diagnostic and therapeutic challenges posed by iMCD TAFRO. In cases with refractory disease despite targeted IL-6 inhibition, additional IL-1 inhibition might pose a further treatment option.

## Introduction

TAFRO syndrome is considered a fulminant, hyperinflammatory manifestation of idiopathic multicentric Castleman’s disease (iMCD TAFRO). The pathognomonic combination of thrombocytopenia, anasarca, fever, reticulin myelofibrosis (or renal insufficiency), and organomegaly have given the disease its name (1–3). The diagnosis is based on clinical signs, symptoms, and specific histopathological findings (3–9). The 5-year survival rate for iMCD TAFRO is approximately 66%, with most deaths occurring within the first few months after diagnosis (3, 5, 10). However, based on the complexity of the diagnosis, the likelihood of underreported mortality is high (1, 2). Although IL-6 inhibition provides a relevant treatment option, the likelihood of sustained remission with this treatment can be achieved in only 30%-50% of patients. Most patients require ongoing therapy to maintain disease control (1, 3, 5).

iMCD TAFRO typically presents with fulminant sepsis-like features, generalized lymphadenopathy and a rapid deterioration (5, 11). Initially, patients are usually treated in the intensive care unit. They are initially treated for fulminant bacterial infections, but if their condition worsens, they often receive immunomodulatory treatment (5, 10, 12, 13). To approach the diagnosis, infections, autoimmune diseases and malignancies with similar clinical presentation must be evaluated. In the light of the diagnosis Castleman disease the most common pathogenetic driver, HHV-8 infection in HIV-positive patients, must be excluded. Further important differential diagnosis in HHV-8 negative cases are cytokine storm syndromes such as hemophagocytic lymphohistiocytosis (HLH) (3). In contrast to iMCD, HLH is associated with significantly higher levels of ferritin and pronounced hemophagocytosis in lymph node and/or bone marrow (3, 4, 9, 14).

In iMCD TAFRO, IL-6 acts as a central proinflammatory mediator. It drives the activation of the PI3K/AKT/mTOR signaling pathway, which correlates with disease severity, capillary leakage, and anasarca and therefore acts as the main therapeutical target (15). However, in approximately 50% of cases, IL-6 inhibition alone does not provide sufficient and sustained treatment success (3, 5, 13). Additional IL-1 inhibition—although justified on pathophysiological grounds and recommended by few experts (3)—has so far only been documented in individual cases (16).

## Patient information and clinical findings

A previously healthy 21-year-old Caucasian man developed in April 2024 diarrhea and abdominal pain. As symptoms progressed, including fever >39 °C, increasing fatigue, and persistent gastrointestinal complaints, he was admitted to a regional hospital. During the initial workup, stool testing confirmed rotavirus infection. Within days, he developed the picture of septic syndrome, which led to the suspicion of bacterial translocation, secondary to viral gastroenteritis. Despite thorough microbiological workup, no relevant pathogen was found. The patient remained febrile, and inflammatory markers continued to rise despite broad-spectrum antimicrobial therapy. Moreover, hemodynamic status and renal function progressively worsened, necessitating vasopressor support and hemodialysis. A CT scan showed generalized lymphadenopathy, splenomegaly, and polyserositis including pleural effusion and ascites. Due to his continuing deterioration despite intensive therapeutic efforts, he was transferred to a tertiary care center.

As the clinical course worsened further, the patient was started on immunosuppressive therapy including pulsed corticosteroids (1 mg/kg prednisolone equivalent/day) and immunoglobulin. The overall presentation then raised suspicion of an underlying hyperinflammatory syndrome, notably hemophagocytic lymphohistiocytosis (HLH) with an H-score of 146 and an overall moderate probability for this diagnosis (14, 17). Based on the suspected HLH, a treatment with the IL-1 receptor antagonist anakinra was initiated (200mg s.c./day). Under this regimen, the patient’s condition improved the corticosteroids could be tapered, allowing step-down to non-intensive care and eventually transfer to rehabilitation in August 2024.

**Figure 1:**
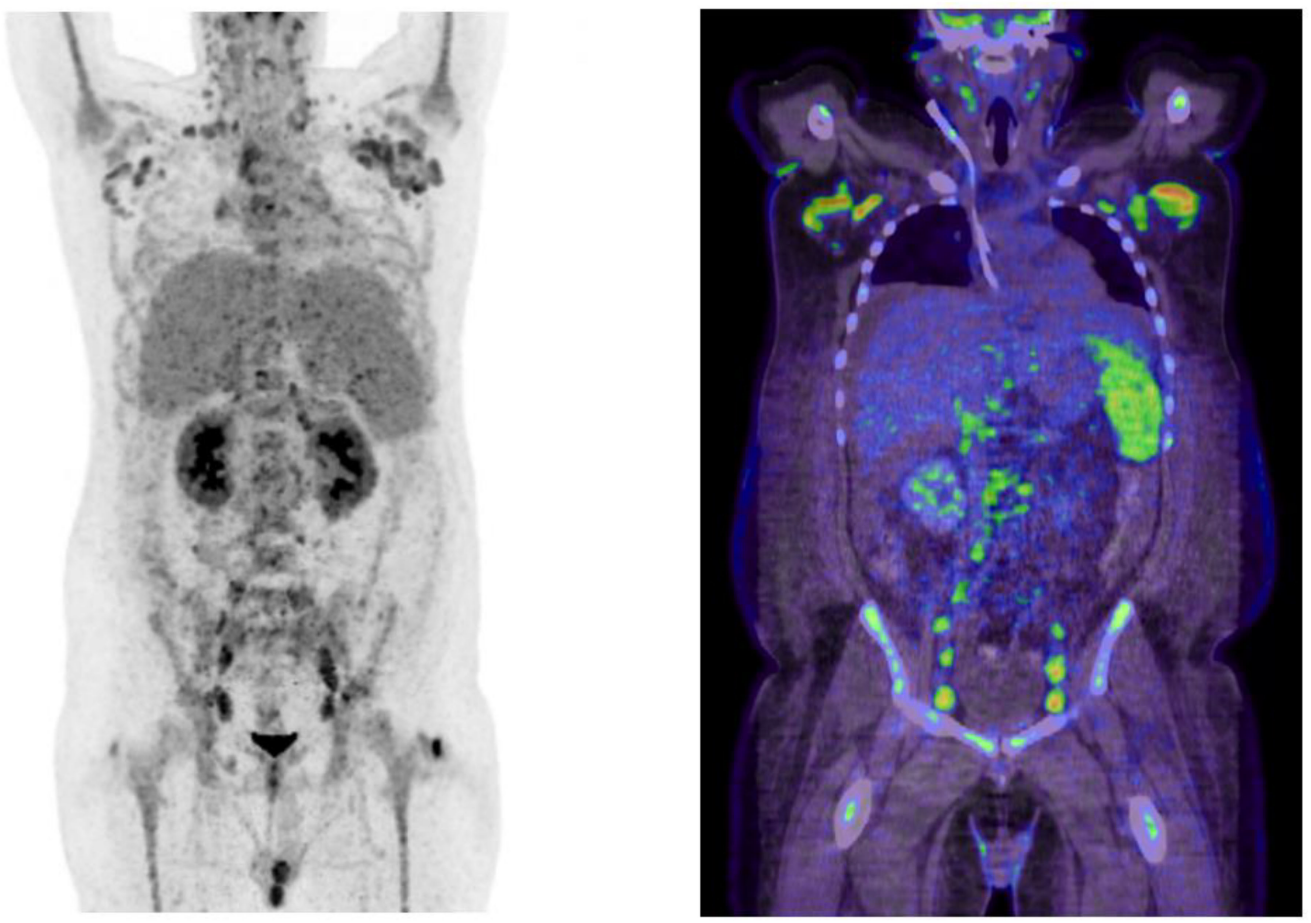
PET CT imaging revealed generalized hypermetabolic lymphadenopathy involving cervical, mediastinal, axillary, abdominal, and inguinal lymph nodes, consistent with multicentric Castleman disease.

**Figure 2:**
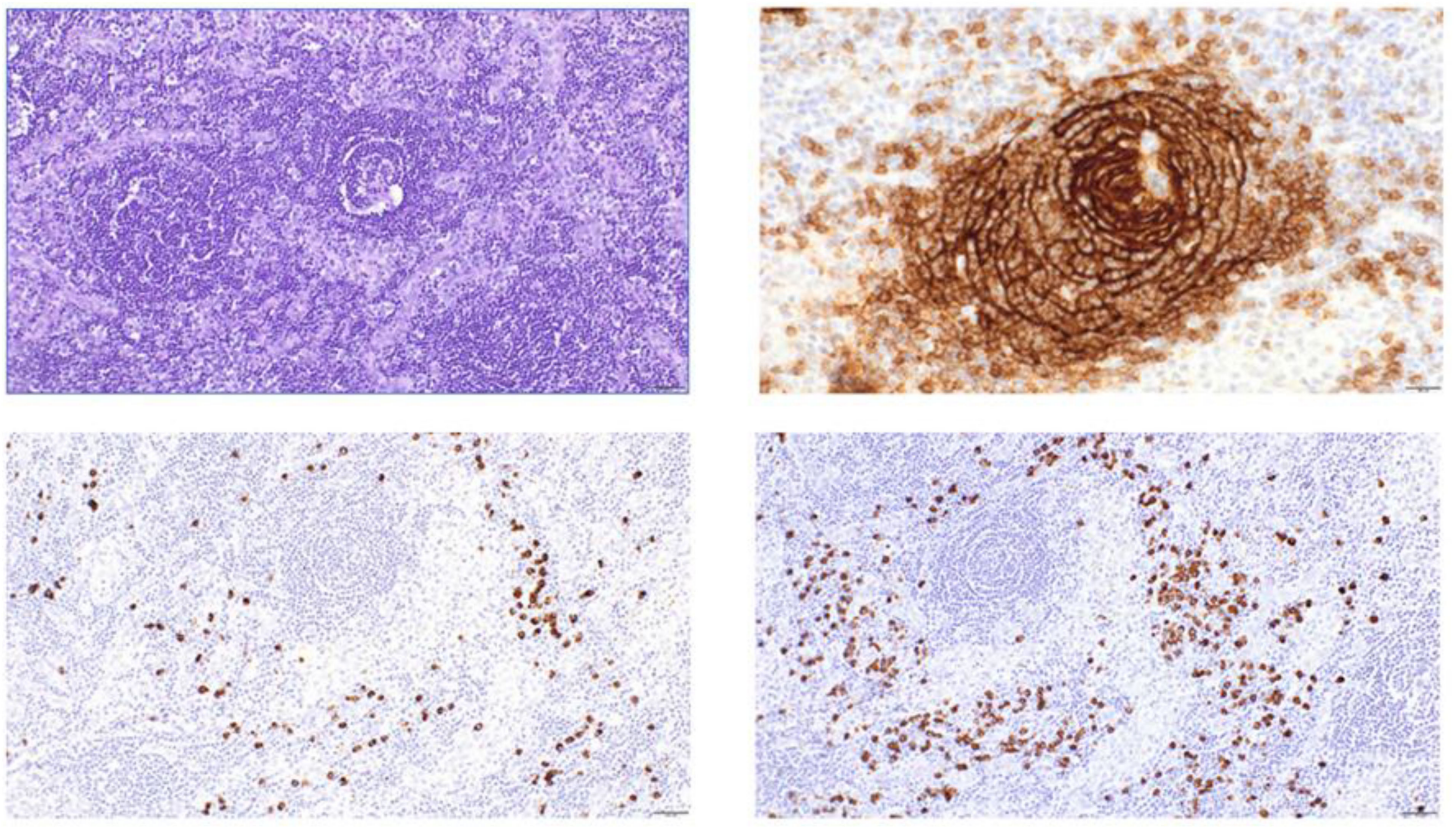
Histopathology of the lymph node revealed regressive changes of the B-cell follicles and an increase in interfollicular small vessels (A, periodic acid-Schiff-staining (PAS) ×200). The regressive B-cell follicles lack germinal centers, and onion-shaped proliferations of the processes of follicular dendritic cells can be observed within the follicle mantle (B, CD21 × 400). An interfollicular increase in polytypic plasma cells is present (C, lambda- ×200 and D, kappa- × 200, *in-situ* hybridization).

**Figure 3:**
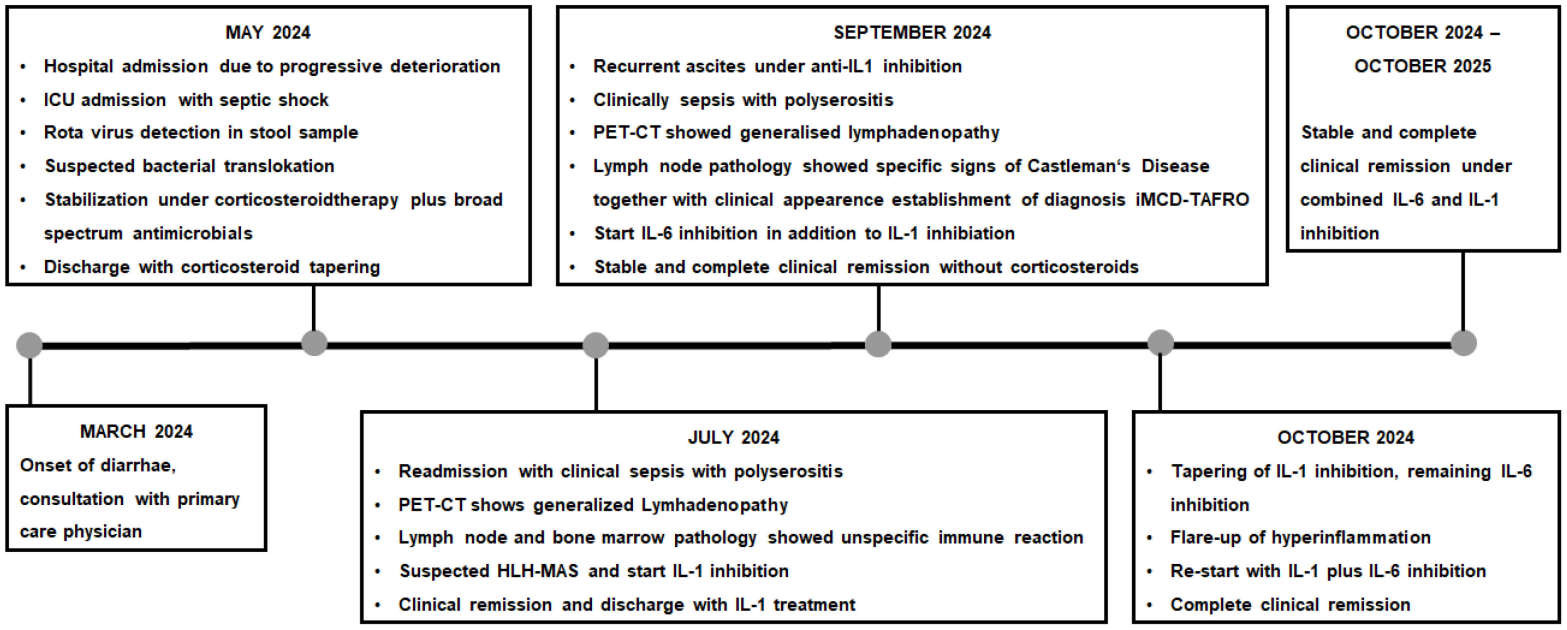
Course of disease from onset of symptoms until complete remission.

Despite initial stabilization, the patient suffered another flare of systemic inflammation 1 month later. This time, the leading symptom was massive ascites necessitating drainage of 16-L sterile ascites within 10days. A PET CT confirmed hypermetabolic lymphadenopathy and splenomegaly (**Figure 1**). An excision of an axillary lymph node was performed and submitted to the National Reference Center for Lymphoma Studies. This time, histopathological analyses revealed pathognomonic signs of leading to the diagnosis iMCD TAFRO (**Figure 2**). **Figure 3** illustrates the timeline of the clinical course of disease, including key diagnostic and therapeutic milestones (**Figure 3**).

## Timeline

## Diagnostic assessment

Laboratory testing showed leukocytosis (25.7 × 10³/μL), anemia (hemoglobin 7.5 g/dL), thrombocytopenia (27 × 10³/μL), CRP 335 mg/dL, impaired renal function (eGFR 22 mL/min), hypoalbuminemia (17.7 g/L), elevated ferritin (3,571 ng/mL), soluble interleukin-2 receptor (sIL-2R, 16,700 IU/mL), interleukin-6 (IL-6, 124pg/mL), and vascular endothelial growth factor (VEGFa, 998pg/mL) but no relevant gammaglobulinemia (**Table 1**). Infectious disease workup, among others HIV, HHV-8, EBV, CMV, toxoplasmosis, tbc, and parvovirus B19, yielded negative results. The workup for autoantibody diseases, notably ANA, anti-ENAs, ANCAs, IgG4, and anti-ds-DNA, showed a negative result. Due to sustained renal dysfunction, transvenous kidney biopsy was performed, demonstrating secondary thrombotic microangiopathy (TMA), compatible with renal involvement in the hyperinflammatory process. A positron emission computed tomography (PET CT) confirmed metabolically active lymphadenopathy in supra- and infradiaphragmatic regions, along with hypermetabolic splenomegaly (**Figure 1**). The patient showed persistent bicytopenia (erythrocytes and thrombocytes); this prompted a bone marrow puncture and lymph node biopsy. The results initially showed signs consistent with reactive inflammation without further specific results.

**Table 1:** Relevant laboratory values.

Laboratory parameter	Result	Normal range
Leukocytes	25.7 × 10³/μL	3.7 – 9.2 × 10³/μL
Hemoglobin	7.5 g/dL	12.0 – 17.9 g/dL
Thrombocytes	27 × 10³/μL	154 – 401 × 10³/μL
CRP	335/mg/dL	<0.5mg/dL
eGFR	22 mL/min	>90 mL/min
Albumin	18 g/L	35-52 g/L
ferritin	3,571 ng/mL	30–400 ng/mL
sIL-2R	16,700 IU/mL	223–710 IU/mL
IL-6	124 pg/mL	<7 pg/mL
VEGFa	998 pg/ml	30–883 pg/mL

Only the repeated lymph node histopathology then revealed grade 2 regressive follicles and reactive plasma cell infiltration (**Figure 2**), fulfilling one major diagnostic criterion for multicentric Castleman disease (MCD). Immunohistochemistry demonstrated preserved follicular dendritic cell networks with few plasma cells expressing IgG and HHV-8 negativity. This time, based on the histopathological results, together with the clinical presentation, the diagnosis of idiopathic multicentric Castleman disease with TAFRO Syndrome (iMCD TAFRO) was established. The diagnosis was based on the fulfillment of 2017 consensus criteria (major criteria: histopathologic lymph node features consistent with the iMCD spectrum plus generalized lymphadenopathy; minor criteria: elevated CRP, anemia, thrombocytopenia, hypoalbuminemia, renal dysfunction, polyclonal hypergammaglobulinemia, B-symptoms, splenomegaly, and polyserositis; exclusion criteria: HHV-8/EBV/CMV/HIV/toxoplasmosis/tbc negativity, exclusion of rheumatological disorder based on overall negative screening for autoantibodies, exclusion of malignant disorders by repeated histopathological examination of enlarged lymph nodes and bone marrow, notably without a sign of hematophagocytosis) (8). Based on the proposed severity score by Masaki et al., the patient reached a score of 11 of 12 being representative for grade 5 (highest severity) (6, 18). Genetic testing included whole exome sequencing (WES) using next-generation sequencing (NGS) and revealed a heterozygous variant of unknown significance in PI3KCD (NC_000001.11:g.9720854C>T; NM_005026.5:c.1634C>T; p.Ala545Val).

## Therapeutic intervention, outcome, and follow-up

Based on the diagnosis of iMCD TAFRO, IL-6 inhibition with tocilizumab (8mg/kg i.v. every 4 weeks) in addition to the established IL-1 blockade (100mg s.c./day) was started. We chose tocilizumab over siltuximab as this agent is routinely applied in our department, which was quickly available and could safely be administered (19). Under this combined therapy, the patient showed rapid and substantial clinical improvement and remained clinically stable with normalizing laboratory values and no clinical signs of disease recurrence. In order to achieve the minimum therapy necessary, we attempted to gradually taper anakinra. Unfortunately, this led to another inflammatory flare-up. After anakinra was reinitiated with the dose of 100mg s.c./day, we observed again rapid recompensation. The patient is still under close and frequent outpatient surveillance without further antimicrobial prophylaxis. However, combined immunotherapy has not shown signs of infection which would justify a general antimicrobial prophylaxis. After more than 12 months of follow-up, he still remains stable and at home.

## Discussion

This case illustrates diagnostic and therapeutic challenges of idiopathic multicentric Castleman disease with TAFRO syndrome (iMCD TAFRO). Our patient exhibited the characteristics of acute life-threatening systemic inflammation with pronounced ascites and generalized lymphadenopathy.

The clinical presentation and initial investigations pointed to non-specific lymphomatous diseases but also to possible cytokine storm syndromes such as hematophagocytic lymphohistiocytosis (HLH). However, the differential diagnosis of HLH remained *a priori* unlikely. Only significantly higher ferritin levels (>10,000 ng/mL) provide a sufficiently specific marker for HLH. Furthermore, histopathological examinations revealed no signs of hemophagocytosis (17). However, we found signs that are representative of the hyaline vascular subtype of Castleman disease (HV). **Table 2** provides a concise comparison of signs and symptoms seen iMCD TAFRO, HLH, and other most relevant differential diagnoses.

**Table 2:** Comparison of diagnostic results in idiopathic multicentric Castleman disease with TAFRO features (iMCD-TAFRO), hemophagocytic lymphohistiocytosis (HLH), and other relevant mimics (such as iMCD-NOS, autoimmune diseases, and lymphoma) (3, 7–9, 14, 17).

Feature	iMCD-TAFRO	HLH	Other mimics (iMCD-NOS, autoimmune, lymphoma)
Clinical presentation	Acute onset; fever; anasarca; abdominal pain; organomegaly (hepatosplenomegaly); may lack prominent lymphadenopathy	Acute onset; fever; hepatosplenomegaly; cytopenias; neurologic symptoms; rash	Subacute/chronic; variable fever; lymphadenopathy; organomegaly; less anasarca
Thrombocytopenia	Marked, often transfusion-resistant	Marked, part of bicytopenia	Mild or absent; iMCD-NOS may have thrombocytosis
Anasarca/edema	Prominent, often with pleural effusions	May occur, but less prominent	Rare; may occur in severe autoimmune disease
Fever	High, persistent	High, persistent	Variable; less common in indolent mimics
Organomegaly	Hepatosplenomegaly, mild lymphadenopathy	Hepatosplenomegaly, lymphadenopathy	Lymphadenopathy prominent in lymphoma/iMCD-NOS
Renal dysfunction	Common, often with proteinuria	May occur (secondary to multiorgan failure)	Variable; less common
Hemophagocytosis	Absent or rare	Characteristic (bone marrow, other tissues)	Absent
Hyperferritinemia	Mild to moderate	Marked, often >10,000 ng/mL	Mild or absent
CRP/ESR	Elevated CRP, variable ESR	Elevated CRP, variable ESR	Elevated in inflammation
IgG/hypergamma-globulinemia	Normal or low IgG; polyclonal gammopathy rare	Normal or low IgG	iMCD-NOS: polyclonal hypergammaglobulinemia
Anti-SSA/Ro antibodies	Often positive	Negative	Negative
Imaging	Mild lymphadenopathy; marked effusions/anasarca; organomegaly	Hepatosplenomegaly; lymphadenopathy; CNS lesions (MRI)	Prominent lymphadenopathy (lymphoma); variable effusions
Bone marrow	Reticulin fibrosis; mild plasmacytosis	Hemophagocytosis; cytopenias	Plasmacytosis (iMCD-NOS); malignant cells (lymphoma)

The important distinction between secondary Castleman disease and idiopathic variants was made by immunohistochemical exclusion of HHV-8 infection (4, 8, 9). Within the entity of iMCD, there are three relevant forms: iMCD TAFRO, iMCD with plasmacytic lymphadenopathy (iMCD IPL), and iMCD not otherwise specified (NOS) (9, 20). The clinical presentation specifically met the prerequisites of iMCD TAFRO (3, 9). In iMCD TAFRO, the lymphohistopathological types HV and mixed (hyaline vascular and plasmatic cells, HV/PC) have been predominantly observed. In contrast to that, iMCD IPL cases appear to be primarily plasma cell (PC) driven, whereas iMCD NOS apparently is a collection of heterogeneous cases, clinically distinct from TAFRO and histopathologically distinct from iMCD IPL (20, 21).

The pathogenesis of iMCD involves a complex interplay of dysregulated cytokine signaling with IL-6 as a central mediator. Whereas in iMCD TAFRO IL-6 is mainly expressed by endothelial cells, in iMCD IPL, plasma cells are the main IL-6 drivers (21). The IL-6 overproduction activates downstream signaling pathways such as PI3K/AKT/mTOR and other immune cells (2, 3). In iMCD TAFRO, a pronounced activation of the PI3K-Akt signaling pathway is associated with increased angiogenesis and vascular permeability (22). Interestingly, *post-hoc* genetic testing of our patient identified a heterozygous variant of unknown significance (VUS) in the phosphatidylinositol‐4,5‐bisphosphate 3‐kinase, catalytic subunit delta gene (PI3KD) (23). Phosphoinositide 3-kinases, particularly the PI3KD pathway, play a crucial role in regulating immune functions affecting, e.g., T- and B-cell function (24). The found VUS in PIK3CD is rare and has not been previously published. Functional data for this variant is not known. Pathogenic variants in PIK3CD are, e.g., associated with activated PI3Kdelta syndrome (APDS) (25). This is a gain-of-function disorder leading to constitutive activation of this specific pathway and downstream hyperactivation of AKT and mTOR signaling. This results in immune dysregulation, lymphoproliferation, and increased risk of lymphoma, as well as recurrent infections and autoimmunity. In IL-6 refractory iMCD, mTOR activation is a recognized molecular signature, with increased phosphorylation of mTOR effectors in affected lymph nodes and peripheral immune cells (15). This hyperactivation is mechanistically plausible given the convergence of cytokine signaling. Thus, mTOR inhibition with sirolimus is an evidence-based option for IL-6-refractory iMCD. Clinical studies demonstrated durable remissions and biochemical improvement in patients who failed anti-IL-6 therapy (26).

The main treatment options in iMCD include high-dose glucocorticoids followed by IL-6 inhibition. Siltuximab is currently the only FDA-approved IL-6 blocker for Castleman disease (19, 27, 28). The progression-free survival associated with this treatment was 15 months (29). However, iMCD TAFRO cases show in about 50% insufficient treatment results under IL-6 inhibition, thus requiring second-line options (2, 3, 5, 11). Second-line treatments are cyclosporine A, rituximab, and cyclophosphamide (3, 10). However, there are no randomized, controlled studies to date. The available evidence for TAFRO is based exclusively on retrospective cohorts (12, 13, 30).

Considering therapeutic strategies and outcomes, there seem to be relevant differences in the studied Japanese and Western iMCD-TAFRO cohorts (10, 13). Japanese patients show higher disease activity and mortality. TAFRO is recognized there as a distinct, severe inflammatory syndrome treated with corticosteroids, tocilizumab, cyclosporin A, or rituximab. In Western countries, iMCD-TAFRO is considered a subtype of iMCD, with siltuximab as standard first-line therapy. A recently published study showed the effectivity of ruxolitinib (JAK1/2 inhibitor) in combination with ropeginterferon alfa-2b. This could be a promising new option for refractory TAFRO-like syndrome. However, further studies are needed to define their optimal use and long-term outcomes (31).

In our case, an earlier initiated IL-1 inhibition led to partial disease. Given his critical condition, we continued this treatment but additionally started IL-6 inhibition. This combination therapy led to the complete remission of symptoms. However, in an attempt to reduce IL-1 inhibition and switch to anti-IL-6 monotherapy, our patient developed a flare-up of symptoms. After resuming treatment, the symptoms completely subsided again, underscoring the importance of the established combination of IL-6 and IL-1 inhibition. There are case reports on the use of IL-1 blockade in patients with iMCD TAFRO as second line (16, 32, 33). IL-1 is known to be a key upstream inducer of IL-6 production through intracellular signaling pathways, also including PI3K/AKT/mTOR (34). However, there are no published studies on combination therapy with IL-1 and IL-6 blockade.

Current adult iMCD guidelines do not provide a formal recommendation for dual IL-1 plus IL-6 blockade in the TAFRO subtype. The use of such a combination therapy is not established in consensus statements or clinical practice standards (3, 11). The available clinical evidence consists primarily of case reports and small series. There, anakinra was seen as a second-line or rescue therapy, most often as monotherapy. For instance, there are some pediatric TAFRO cases with successful high-dose intravenous administration, in patients refractory to IL-6 blockade (16). Mechanistically, there is plausibility for dual blockade: IL-1 can upregulate IL-6, and both cytokines converge on the PI3K/AKT/mTOR pathway, which is implicated in the pathogenesis of IL-6-blockade-refractory iMCD TAFRO (15).

## Conclusion

This case illustrates the diagnostic and therapeutic challenges of idiopathic multicentric Castleman disease with TAFRO syndrome (iMCD TAFRO). Treatment with IL-1 blockade initially provided partial symptom control, but sustained remission was achieved only after combination therapy including additional IL-6 inhibitors. The patient’s complete remission demonstrates the efficacy of dual IL-6 and IL-1 inhibition in refractory cases of iMCD TAFRO. Further studies are necessary to prove this concept in other treatment refractory cases.

## Data Availability

The original contributions presented in the study are included in the article/supplementary material. Further inquiries can be directed to the corresponding author.
