# Correction: TAFRO syndrome requiring combined IL 6 and IL 1 inhibition: a case report

**DOI:** 10.3389/fimmu.2026.1786154

**Published:** 2026-01-23

**Authors:** Frontiers Production Office

**Keywords:** case report, combined IL-6 and IL-1 inhibition, idiopathic multicentric Castleman disease, iMCD, TAFRO syndrome

There was a mistake in **Table 2** as published. An incorrect file was erroneously published in place of **Table 2**. The corrected **Table 2** appears below.

**Table 2:** Comparison of diagnostic results in idiopathic multicentric Castleman disease with TAFRO features (iMCD-TAFRO), hemophagocytic lymphohistiocytosis (HLH), and other relevant mimics (such as iMCD-NOS, autoimmune diseases, and lymphoma) (3, 7–9, 14, 17).

Feature	iMCD-TAFRO	HLH	Other Mimics (iMCD-NOS, Autoimmune, Lymphoma)
Clinical Presentation	Acute onset; fever; anasarca; abdominal pain; organomegaly (hepatosplenomegaly); may lack prominent lymphadenopathy	Acute onset; fever; hepatosplenomegaly; cytopenias; neurologic symptoms; rash	Subacute/chronic; variable fever; lymphadenopathy; organomegaly; less anasarca
Thrombocytopenia	Marked, often transfusion-resistant	Marked, part of bicytopenia	Mild or absent; iMCD-NOS may have thrombocytosis
Anasarca/Edema	Prominent, often with pleural effusions	May occur, but less prominent	Rare; may occur in severe autoimmune disease
Fever	High, persistent	High, persistent	Variable; less common in indolent mimics
Organomegaly	Hepatosplenomegaly, mild lymphadenopathy	Hepatosplenomegaly, lymphadenopathy	Lymphadenopathy prominent in lymphoma/iMCD-NOS
Renal Dysfunction	Common, often with proteinuria	May occur (secondary to multiorgan failure)	Variable; less common
Hemophagocytosis	Absent or rare	Characteristic (bone marrow, other tissues)	Absent
Hyperferritinemia	Mild to moderate	Marked, often >10,000 ng/mL	Mild or absent
CRP/ESR	Elevated CRP, variable ESR	Elevated CRP, variable ESR	Elevated in inflammation
IgG/Hypergamma-globulinemia	Normal or low IgG; polyclonal gammopathy rare	Normal or low IgG	iMCD-NOS: polyclonal hypergammaglobulinemia
Anti-SSA/Ro Antibodies	Often positive	Negative	Negative
Imaging	Mild lymphadenopathy; marked effusions/anasarca; organomegaly	Hepatosplenomegaly; lymphadenopathy; CNS lesions (MRI)	Prominent lymphadenopathy (lymphoma); variable effusions
Bone Marrow	Reticulin fibrosis; mild plasmacytosis	Hemophagocytosis; cytopenias	Plasmacytosis (iMCD-NOS); malignant cells (lymphoma)

The original version of this article has been updated.

